# Synthesis and Characterization of Multilayered Diamond Coatings for Biomedical Implants

**DOI:** 10.3390/ma4050857

**Published:** 2011-05-09

**Authors:** Leigh Booth, Shane A. Catledge, Dustin Nolen, Raymond G. Thompson, Yogesh K. Vohra

**Affiliations:** 1 Department of Biomedical Engineering, The University of Alabama at Birmingham, Birmingham, AL 35294, USA; E-Mail: lbooth@uab.edu (L.B.); 2 Department of Physics, the University of Alabama at Birmingham, Birmingham, AL 35294, USA; E-Mail: catledge@uab.edu (S.A.C.); 3 Vista Engineering, Birmingham, AL 35203, USA; E-Mails: dnolen@vistaeng.com (D.N.); rthompson@vistaeng.com (R.G.T.)

**Keywords:** nanocrystalline diamond, chemical vapor deposition, multilayer, adhesion

## Abstract

With incredible hardness and excellent wear-resistance, nanocrystalline diamond (NCD) coatings are gaining interest in the biomedical community as articulating surfaces of structural implant devices. The focus of this study was to deposit multilayered diamond coatings of alternating NCD and microcrystalline diamond (MCD) layers on Ti-6Al-4V alloy surfaces using microwave plasma chemical vapor deposition (MPCVD) and validate the multilayer coating’s effect on toughness and adhesion. Multilayer samples were designed with varying NCD to MCD thickness ratios and layer numbers. The surface morphology and structural characteristics of the coatings were studied with X-ray diffraction (XRD), Raman spectroscopy, and atomic force microscopy (AFM). Coating adhesion was assessed by Rockwell indentation and progressive load scratch adhesion tests. Multilayered coatings shown to exhibit the greatest adhesion, comparable to single-layered NCD coatings, were the multilayer samples having the lowest average grain sizes and the highest titanium carbide to diamond ratios.

## 1. Introduction

The generation of wear debris from the articulating surfaces of total joint replacements can lead to a variety of complications, including inflammation, osteolysis, and aseptic loosening at the implant site [1,2,3]. A potential solution is to decrease the wear debris by applying nanocrystalline diamond (NCD) coatings to the articulating surfaces to reduce component wear, thus reducing complications. NCD coatings produced by microwave plasma chemical vapor deposition (MPCVD) exhibit many desirable properties such as excellent wear resistance, minimal surface roughness, and chemical inertness which are favorable to orthopaedic implants. Previous studies of NCD coated Ti-6Al-4V disks have demonstrated reduced wear rates *versus* CoCrMo alloys [4]. Furthermore, an advantage of CVD diamond coatings would be their biocompatibility, comparable to other metals and alloys commonly used in implantable devices [5,6]. 

NCD coatings on Ti-6Al-4V substrates synthesized by MPCVD with methane, hydrogen, and nitrogen plasma chemistry have been previously reported to exhibit good adhesion [7,8]. These coatings featured excellent surface roughness (14–30 nm) and high hardness (70–90 GPa). Additionally, ultrananocrystalline diamond (UNCD) coatings synthesized under a helium rich plasma was shown to further reduce grain size (5–6 nm) and surface roughness (9–10 nm) while maintaining hardness in the range of 56–72 GPa [9,10]. Previous studies of multilayered CVD diamond coatings on temporomandibular joint components demonstrated enhanced wear characteristics *versus* single-layered NCD coatings [11]. The application of NCD coatings applied to titanium implants could potentially improve fatigue and abrasion performance [12].

There is a great amount of interest in the application of multilayer coatings to improve bulk coating or thin film toughness and adhesion [13,14,15,16,17]. Multilayer coatings consisting of two or more layers with unique properties have been associated with increased resistance to crack propagation by acting to deflect cracks at the interfaces of separate layers [16,17]. Furthermore, multilayered coatings have been shown to improve the adhesive properties of a variety of coating systems [15,18]. Therefore, the interest in synthesizing multilayered CVD diamond coatings with alternating layers of NCD and microcrystalline diamond (MCD) would be to potentially improve upon the toughness or adhesive properties of single-layered NCD coatings while still maintaining high hardness and low surface roughness. 

This study describes the synthesis of multilayered diamond coatings of alternating NCD and MCD layers and UNCD surface layer on Ti-6Al-4V substrates by MPCVD. The number of layers and relative thickness ratios of NCD to MCD were varied and effects on coating structure and surface topography were evaluated by X-ray diffraction (XRD), Raman spectroscopy, and atomic force microscopy (AFM). Adhesion of the various coatings was also assessed and compared by Rockwell indentation and progressive load scratch adhesion tests. 

## 2. Methods and Materials

### 2.1. Coating Synthesis

Ti-6Al-4V alloy disks with diameter of 7 mm diameter and approximate thickness of 1 mm were fabricated from stock sheet of Ti-6Al-4V (Robin Materials, Mountain View, CA) and polished to a mirror finish. Substrates were cleaned with a series of acetone, methanol, and water followed by mechanical seeding with a 2–4 µ diamond powder on a napped polishing cloth. A 6 kW MPCVD reactor (Wavemat, Ann Arbor, MI) was used to deposit NCD, MCD, UNCD, and multilayered diamond coatings on polished substrates. Reaction gases were delivered at flow rates of 500 sccm (H_2_), 88 sccm (CH_4_) and 8.8 sccm (N_2_) for NCD coatings; 500 sccm (H_2_) and 26 sccm (CH_4_) for MCD coatings; and 213 sccm (He), 87 sccm (H_2_), 36 sccm (CH_4_), and 14.4 sccm (N_2_) for UNCD coatings. All depositions were maintained at a pressure of 40 torr and microwave power of 0.82 ± 0.10 kW providing an average substrate temperature of 700 ± 30 °C. A two-color infrared pyrometer was used to plot substrate temperature and determine *in situ* growth rates [19]. 

**Figure 1:**
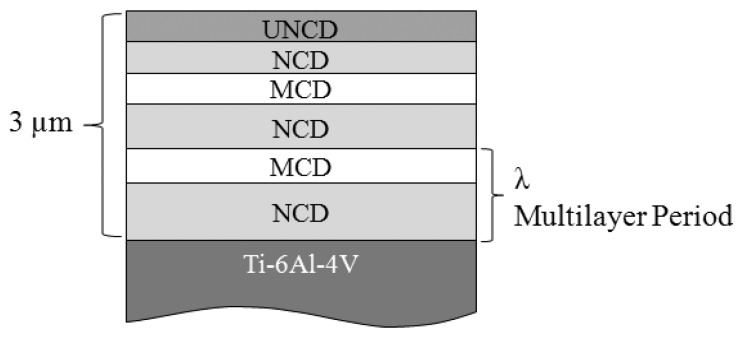
Schematic diagram of multilayered coating structure parameters in this study.

Multilayer coatings were deposited by varying the plasma chemistry at appropriate time intervals to achieve the desired layer thickness. Multilayer coating parameters, listed in Table 1, were produced by varying the total number of layers and the relative thickness NCD/MCD ratios. A general coating schematic is shown in Figure 1. Each coating was described as having a multilayer period, λ, defined as the basic repeating NCD/MCD unit. The total coating thickness was maintained at 3 μm for every coating, with basic repeating NCD/MCD units (λ) and a surface layer unit of 150 nm NCD/150 nm UNCD as the final two layers. Each coating configuration was produced in triplicate. 

**Table 1:** Summary of multilayered coating parameters used in this study.

					Thickness (nm)		
Multilayer	λ (nm)	n of λ	Total layers	NCD/MCD	NCD/MCD	NCD/UNCD	Total
M1	900	3	8	3:1	675/225	150/150	3,000
M2	900	3	8	1:1	450/450	150/150	3,000
M3	900	3	8	1:3	225/675	150/150	3,000
M4	300	9	20	1:1	150/150	150/150	3,000
M5	2000	1	4	1:1	1000/1000	850/150	3,000

### 2.2. Coating Characterization

Glancing angle X-ray diffraction (XRD) was used to characterize the crystalline structure of the coatings (X’pert MPD, Philips, Eindhoven, Netherlands). XRD scans were performed across a range of 2*θ* = 20°−80° at a scan speed of 0.013° per second and step size of 0.040°. Short range XRD scans were performed across the 2*θ* = 39°–46° range to evaluate the integrated intensities and full width at half maximums (FWHM) of Ti, TiC, and (111) diamond peaks at approximately 2*θ* = 40°, 42°, and 44°, respectively. Diamond grain size was calculated based on broadening of the (111) diamond peak associated with increased nanometer scale features of the diamond coating using the Scherrer equation. 

Relative sp^3^ and sp^2^ carbon bonding and structural quality of NCD, MCD, and multilayer coatings were examined using micro-Raman spectroscopy. A 514.5 nm argon ion laser source at 100 mW power was focused on the surface of each sample through the 100 X objective of an optical microscope and the scattered signal was analyzed by a high resolution spectrometer (1 cm^−1^ resolution) coupled to a CCD system. A linear baseline was subtracted for each spectral scan to facilitate peak identification and evaluation. 

The surface topography of the coated samples was imaged for comparison using a Topometrix Explorer AFM (Veeco, Santa Barbara, CA) over 5 μm × 5 μm areas. All images were captured in contact mode and processed with a second order leveling and left shading. TopoMetrix SPMLab software was used to analyze average (R_a_) and root mean square (RMS) roughness values for each coating. RMS surface roughness values were used for coating comparisons due to greater significance to variations from the center-line mean [20]. 

Nanoindentation was performed on M1, M3 and NCD samples using a Nanoindenter XP system (MTS Systems, Oak Ridge, TN) with a continuous stiffness (CSM) attachment to evaluate hardness and elastic modulus. These particular multilayered samples were chosen for comparison since they represented the two extreme ratios of NCD/MCD (3:1 and 1:3, respectively). Five separate indentations were performed on each sample with a Berkovich diamond indenter up to a maximum depth of 300 nm (limiting the indentation depth to approximately 1/10th of the coating thickness to reduce substrate effects). A silica standard was indented as well to confirm that blunting of the tip did not occur. Hardness and elastic modulus were determined after unloading using the Oliver and Pharr approach [21]. 

### 2.3. Assessment of Coating Adhesion

Spherical indentation using a standard Rockwell hardness tester was performed on multilayer and NCD coatings as an initial qualitative comparison of coating adhesion. A 1/8 inch diameter tungsten carbide sphere was used to apply loads of 60 kg and 100 kg to the coated surfaces resulting in plastic deformation of the coating with the underlying substrate. Scanning electron microscopy (SEM) was used to analyze indentations for qualitative comparison of resultant perimeter cracking for each coating. 

Progressive load scratch adhesion tests were performed on samples M1 and M4, based on their performance in the Rockwell indentation tests, and compared to NCD coated samples. The coatings were deposited on 15 mm diameter Ti-6Al-4V substrates replicating the 7 mm diameter sample parameters. Scratch tests were performed with a commercial diamond stylometer (Romulus IV, Quad Group, Inc., Spokane, WA, USA). All tests were performed with a 533 µm radius spherical diamond tip, a maximum load of 60 N, displacement of 6 mm, and a load rate of 2 N/s. The tip radius was selected to inhibit blunting of the diamond tip during testing [22]. Scratch tracks were examined microscopically and the critical normal load associated with the onset of cracking or cohesive failure of the coating (LC_1_) and adhesive failure of the coating-substrate (LC_2_) were determined.

## 3. Results and Discussion

### 3.1. Coating Characterization

**Figure 2:**
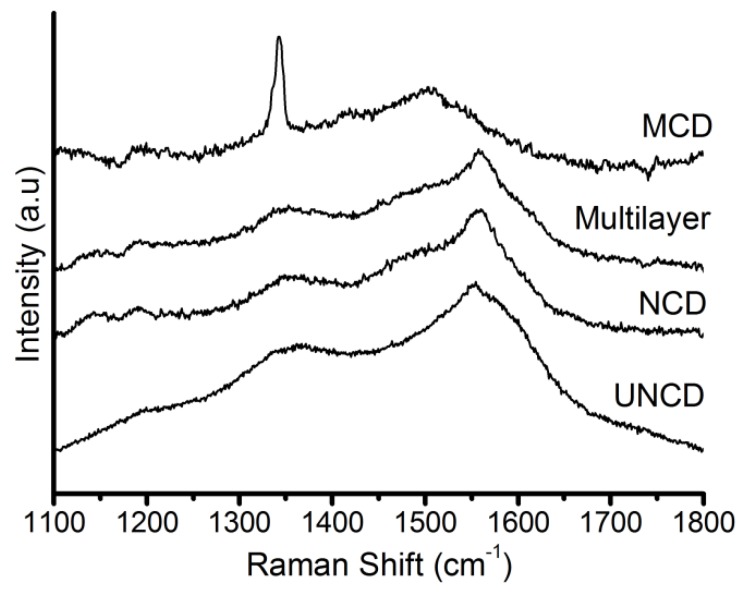
Typical micro-Raman spectra of NCD, MCD, UNCD, and multilayer coatings.

Micro-Raman spectroscopy, shown in Figure 2, was used to compare carbon bonding states of single-layered MCD, NCD, UNCD, and multilayered coatings. MCD coatings exhibited a well-defined peak near 1,332 cm^−1^ that may be associated with more ordered, crystalline diamond. Minimal distinguishable differences were exhibited by the Raman spectra of NCD, UNCD, and multilayer coatings. Peak broadening in the 1,550 cm^−1^ region may be associated with more amorphous carbon bonding typical of nanocrystalline CVD diamond coatings [23]. Since the top 300 nm of every multilayer coating is consistent (150 nm NCD and 150 nm UNCD), this would explain the indistinguishable variation in Raman spectra of multilayer samples. 

**Figure 3:**
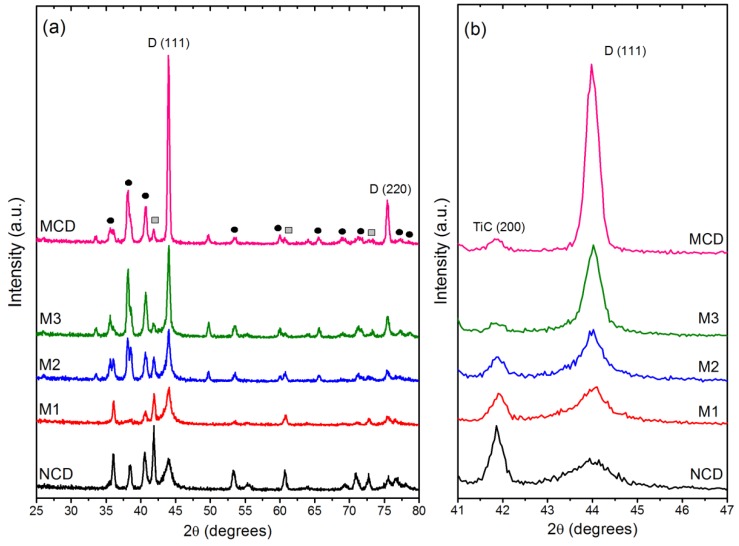
(**a**) XRD pattern of NCD, MCD, and multilayered coatings (λ = 900 nm); (**b**) Shorter range scan highlighting variation in TiC (200) and D (111) reflections for NCD, MCD, and multilayered coatings.

**Figure 4:**
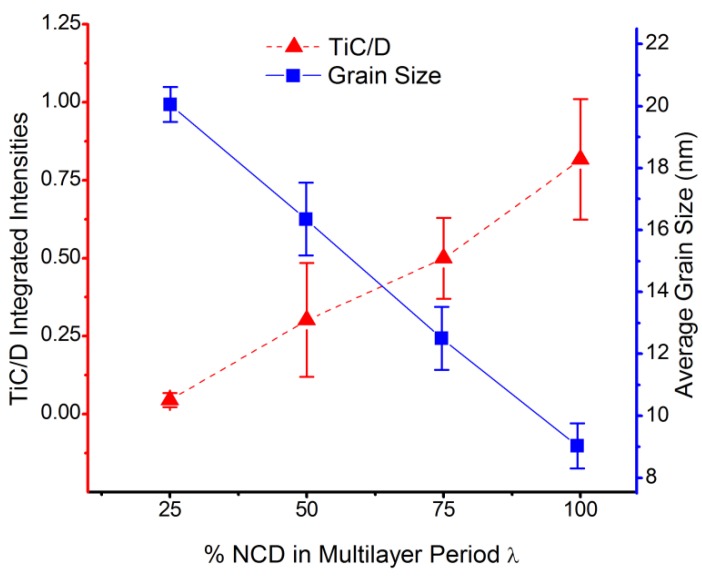
TiC/D ratio and average diamond grain size for multilayered coatings with varying percentage of NCD in multilayer period λ.

Analysis of XRD patterns of multilayer coatings further revealed differences in crystalline features. Broadening of the (111) diamond peak with increasing NCD component may be seen in Figure 3a. Evaluation of grain size, based on the Scherrer equation and FWHM of the (111) diamond peak, revealed increasing nanocrystalline features of multilayer coatings with increased NCD content. A noticeable intensity variation of the TiC peaks was observed for the various coatings synthesized in this study (Figure 3b). General trends of increased TiC/D integrated intensity and decreasing diamond grain size with increased NCD content may be readily seen in Figure 4. Since TiC is primarily associated with the coating/substrate interface, it is probable that high ratios of TiC/D could be indicative of increased adhesion to Ti alloy substrates. 

**Figure 5:**
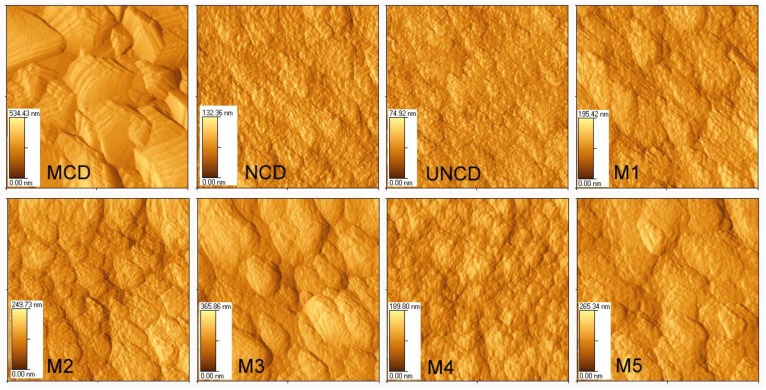
AFM images of various coatings surfaces, revealing differences in surface topography of multilayered coatings in comparison to single-layered MCD, NCD, and UNCD coatings.

**Figure 6:**
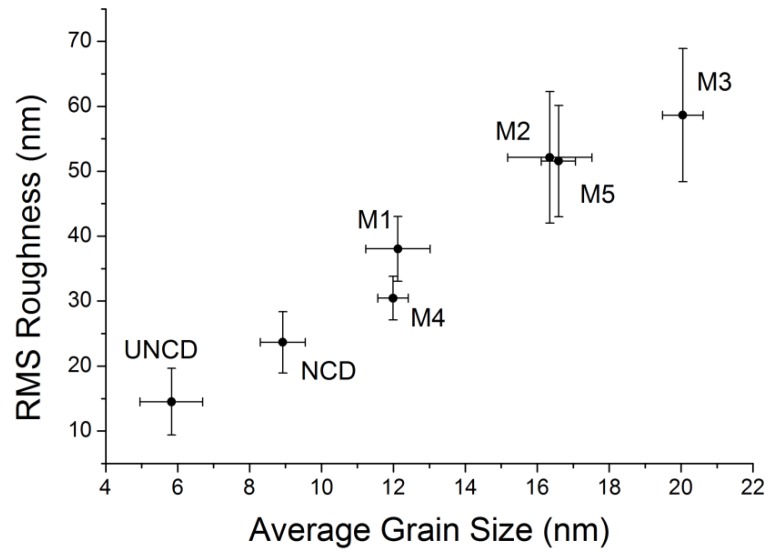
Average RMS surface roughness as related to average diamond grain size for multilayered, NCD, and UNCD coatings.

Atomic force microscopy revealed differences in morphology and surface roughness of multilayer, NCD, MCD, and UNCD samples. Surface roughness and grain size for NCD and UNCD samples shown in Figure 5 were comparable to those previously reported [7,9]. Multilayer coatings with increased MCD layer thickness (such as M3 and M5) demonstrated more crystalline morphology and increased surface roughness values. The UNCD top layer did not result in consistent surface morphology and surface roughness of the multilayer coatings, but rather conformed to the features of the underlying coating. An increasing trend of RMS surface roughness *versus* average coating grain size can be established, as depicted by Figure 6. Thus, in the design of multilayered CVD diamond coatings produced from a single deposition process, an important consideration is the effect on surface finish due to MCD component thickness. With regard to biomedical implant applications, it is desirable for the surface finish to be smooth, with roughness values below 50 nm [24]; thus, the multilayered coatings with lowest average surface roughness values (M1 and M4) may have the most potential as wear-resistant coatings.

Nanoindentation of NCD and multilayer coatings revealed moderate differences in hardness and elastic modulus. Hardness for NCD coating was determined to be 68.7 ± 11.5 GPa and elastic modulus was 470.7 ± 38.5 GPa. Multilayers M1 and M3 were chosen for comparison since they were considered to be the two extreme cases for multilayer samples (3:1 NCD/MCD and 1:3 NCD/MCD ratios, respectively). For M1, nanoindentation hardness was 78.9 ± 5.3 GPa and modulus was 565.3 ± 31 GPa while for M3 hardness and modulus were 65.2 ± 3.2 GPa and 516 ± 48.2 GPa, respectively. Hardness and modulus of silica standards used before and after indentations were 9.7 ± 1 GPa and 72.2 ± 5.5 GPa. Limited variation in hardness and moduli data for multilayer coatings in comparison to NCD coatings may be attributed to the 300 nm penetration depth, which indented the same top two layers of each sample (150 nm NCD/150 nm UNCD). Furthermore, the underlying multilayered coating configuration does not appear to significantly affect the measured hardness and modulus of the top surface regions.

### 3.2. Coating Adhesion

**Figure 7:**
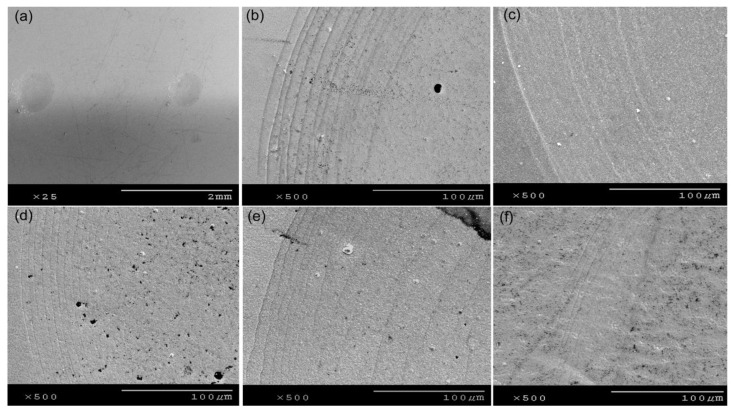
SEM images showing extent of circumferential cracking on (**a**) NCD with 60 kg and 100 kg loads, and 100 kg loads of (**b**) NCD; (**c**) M1; (**d**) M4; (**e**) M3; and (**f**) M2.

Rockwell indentations performed on multilayered coatings and single-layered NCD coatings revealed circumferential microcracks concentrated around the edge of the indentation imprint. This type of cracking may be associated with the significant tensile radial strains produced in the coating and underlying substrate during spherical indentation [25]. All NCD coatings remained adhered to the substrate after 60 kg and 100 kg loads were applied; furthermore, all samples of M1 and M4 remained adhered to the substrates and exhibited similar cracking behavior surrounding the indentation imprint. Multiple samples of multilayered coatings M2, M3, and M5 delaminated at 100 kg loads. Figure 7 depicts images of remaining impressions following Rockwell indentation of single-layered NCD and multilayer coatings. 

In general, NCD, M1, and M4 samples showed evidence of smooth, evenly-spaced circumferential cracks surrounding the impression. Furthermore, samples of M2 and M3 that remained intact after indentation showed circumferential cracks with jagged crack extension, indicating a more brittle mode of fracture. This may be attributed to larger diamond grain size associated with these multilayer coatings. Additionally, it is probable that the reduction in the relative integrated intensities of TiC/D for these multilayer coatings with thicker MCD layers (M2, M3, and M5) could be attributed to the reduced performance of these samples. Of the multilayer coatings, M1 and M4 (which both consisted of relatively thinner layers of MCD, 225 nm and 150 nm, respectively) exhibited the lowest average diamond grain size, lowest surface roughness, and qualitative adhesion similar to that of single-layered NCD samples. 

**Figure 8:**
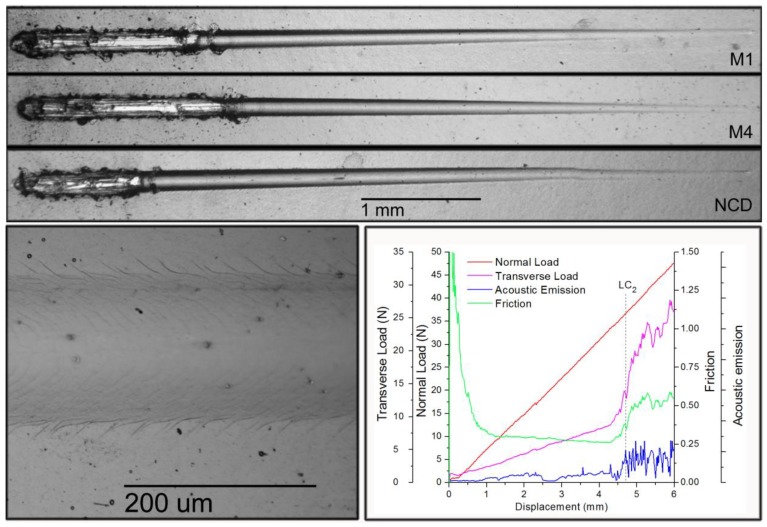
(**a**) Representative scratch track of M1, M4, and NCD samples tested on Ti-6Al-4V substrates; (**b**) Higher magnification of NCD sample showing forward tensile cracking attributed to cohesive failure in coating; (**c**) Sample data output for NCD coated sample.

Progressive load scratch adhesion tests were used as a complementary technique to study the practical adhesion strength of multilayered coatings M1 and M4 in comparison to single-layered NCD coatings. Critical loads associated with cohesive failure (LC_1_) and adhesive failure (LC_2_) are summarized in Table 2 (average ± standard deviation for each sample set). Overall, the multilayered coatings M1 and M4 behaved similarly to single-layer NCD coatings. A typical data set for a single scratch test, depicted in Figure 8(c), includes output for applied normal load, transverse load, friction, and acoustic emission over the displacement of the scratch test. The critical load associated with adhesive failure (LC_2_) consistently coincided with abrupt increases in friction and acoustic emission as the coating began to delaminate from the substrate and the diamond stylus continued to plough through the titanium alloy substrate. It may be noted that extensive delamination and chipping extending from the stylus contact region was not seen, indicating a high level of adhesion between the coating and substrate. Furthermore, it may be noted that for the diamond coatings tested in these experiments, the acoustic emission sensitivity was not sensitive enough to detect the initiation of coating microcracks at loads less than that of LC_2_. Therefore, the critical load associated with the onset of cohesive failure within the coatings (LC_1_) could only be detected by optical examination of the scratch track after testing.

**Table 2:** Summary of critical loads for cohesive failure (LC_1_), and adhesive failure (LC_2_), for multilayered coatings M1 and M4 and single-layered NCD coatings.

Coating	LC_1_ (N)	LC_2_ (N)
M1	12.6 ± 1.4	33.8 ± 2.9
M4	11.2 ± 2.4	32.5 ± 4.4
NCD	14.9 ± 3.7	38.7 ± 3.1

A typical scratch track for each sample tested is shown in Figure 8(a), while increased magnification of the cohesive failure mode that may be seen in NCD samples is depicted in Figure 8(b). These forward chevron tensile cracks tend to be associated with cohesive failure of the coating prior to adhesive failure of the coating-substrate system [26]. Since the coating deforms with the ductile substrate, large bending stresses are induced at the sides of the scratch track, resulting in tensile cracks emerging from the edges of the scratch [27]. Similar modes of cohesive failure were seen for both M1 and M4 multilayer coatings, indicating that the cracking behavior was not distinguishable between multilayer and NCD coatings. All coatings were able to undergo extensive plastic deformation with the underlying substrate prior to adhesive failure. Comparison of adhesive failure loads in Table 2 show that single-layered NCD coatings exhibited a greater level of adhesion than multilayered coatings M1 or M4. Additionally, NCD coatings showed the highest average values for cohesive failure loads (LC_1_); however, variance overlap in the data prevented the conclusion that NCD coatings are associated with increased cohesive failure loading over multilayer coatings M1 and M4. 

## 4. Conclusions 

This study describes the synthesis and characterization of multilayered diamond coatings with alternating NCD/MCD layers on Ti-6Al-4V substrates by microwave plasma CVD processing. Using a constant coating thickness (3 μm), the relative ratio of NCD/MCD and number of layers were varied, and the subsequent effects on coating morphology and structural properties were studied. Increased NCD content for a given multilayer period λ was shown to correlate with decreased average diamond grain size and surface roughness, as well as increased nanocrystallinity based on broadening of (111) diamond reflections from XRD. Multilayered coatings with the lowest average diamond grain sizes and surface roughness (M1 and M4) were shown to perform comparably to single-layered NCD coatings in qualitative Rockwell indentation tests and progressive load scratch adhesion tests. Furthermore, multilayered coatings with higher ratios of TiC/D performed better in Rockwell indentation tests, comparable to single-layered NCD coatings (with highest overall ratio of TiC/D). Therefore, the ratio of TiC/D does seem to correlate with diamond coating adhesion to the Ti-6Al-4V substrate. This would be important to consider in the design of wear-resistant coatings for biomedical implant applications, where coating adhesion to the substrate material would be a primary concern. Progressive load scratch adhesion tests indicated a high level of adhesion for multilayered coatings M1 and M4 and single-layered NCD coatings, with single-layered NCD coatings on average having the highest critical load values to initiate both cohesive failure (tensile cracking) and adhesive failure (delamination from substrate). Future work may include more advanced wear simulator studies to more closely replicate *in vivo* loading and physiological conditions, providing additional analysis and understanding of multilayered diamond coating performance in comparison to single-layered NCD coatings for biomedical implant applications.
